# Understanding Global Deprescribing Policy: Opportunities and Challenges

**DOI:** 10.1093/geroni/igab046.3034

**Published:** 2021-12-17

**Authors:** Catherine Kim, Allison Thompson, Grace Kim, Zachary Marcum, Michelle Keller, Andrea Sorensen, Nicole Brandt

**Affiliations:** 1 University of Maryland School of Pharmacy, BALTIMORE, Maryland, United States; 2 University of Maryland School of Pharmacy, University of Maryland School of Pharmacy, Maryland, United States; 3 University of Washington, Seattle, Washington, United States; 4 Cedars Sinai, Cedars Sinai, California, United States; 5 UCLA, UCLA, California, United States

## Abstract

The landscape of deprescribing, the planned process of dose reduction or stoppage for medications which are no longer of benefit, has been rapidly expanding with global efforts and the formation of regional and national deprescribing networks. The purpose of this qualitative study is to describe successes and challenges about deprescribing from thought-leaders across the world to inform future policy initiatives. We aim to conduct at least 15 key informant (KI) interviews; we have completed 13 to date. Codes were constructed to identify themes that depict the perspectives regarding deprescribing policy across the globe. The KIs primarily represent the fields of pharmacy and medicine from four global regions with years of deprescribing experience ranging from 5 to > 20. We identified two emerging overarching themes through our qualitative analysis: Regional Organization Support and Evidence & Knowledge gaps. Within these overarching themes, we further identified sub-themes and their representative quotes: Network Structure: “idea of the network was threefold: 1) To try and figure out what we need to activate healthcare providers to deprescribe; 2) To do work with community-dwelling seniors to motivate them and give them opportunities to deprescribe; 3) ...getting pharmacists to provide the education to the patients.” Cost-effectiveness: “If we can show that it is cost-effective to deprescribe, that there is actually a return here, not just in health terms but in monetary terms, I think that would really push it along.” This research will help to advance global efforts to optimize medication management.

